# The quality of life of Hungarian adolescents in the light of their emotions

**DOI:** 10.1192/j.eurpsy.2024.743

**Published:** 2024-08-27

**Authors:** N. Rábavölgyi, Z. Mayer, B. Szabó, M. Miklósi

**Affiliations:** ^1^Department of Developmental and Clinical Child Psychology, Eötvös Loránd University Institute of Psychology; ^2^ Eötvös Loránd University Doctoral School of Psychology; ^3^Department of Clinical Psychology, Semmelweis University; ^4^Mentalhygienic Centre, Heim Pál National Pediatric Institue, Budapest, Hungary

## Abstract

**Introduction:**

Mental health professionals pay particular attention to adolescents, as many psychiatric disorders begin at this age, and the mental state of adolescents has been deteriorating worldwide in the last decade. Based on previous international research, the ability to regulate negative emotions and mentalizing - that is, the ability to identify the thoughts and emotions behind one’s own and others’ behaviour - mediate the negative effects of attachment difficulties experienced in close relationships on the quality of life. This relationship has not yet been investigated among Hungarian adolescents. Adolescent events can have a long-term effect on a person’s mental health, so it is very important to examine the factors that influence the quality of life.

**Objectives:**

This research aimed to examine the relationship between attachment, mentalizing, emotion regulation and quality of life among adolescents between 14 and 18 years of age.

**Methods:**

In our non-clinical cross-sectional research, 141 adolescents filled out the Experiences in Close Relationships questionnaire, the Difficulties in Emotion Regulation Scale, the Reflective Functioning Questionnaire and the Quality of Life Scale after informed consent. We tested two mediator models, in which emotion regulation and mentalizing were the mediating variables in the relationship between attachment difficulty and quality of life.

**Results:**

In our analyses, attachment difficulties (*c’* = -1,87, *p* < .001, β = -0.41) and emotion regulation problems (*b* = -0.08, *p* < .001, β = -0.39) also predicted a reduced quality of life. Attachment problems also reduce the quality of life of young people through emotional regulation difficulties (∑ab = -0,81 [-1,21 – -0,45], β = -0.17). However, mentalizing was not significantly related to the adolescents’ quality of life (*b =* -0,05, *p* = .10, β = -0,11). Mentalizing also did not mediate the relationship between attachment and quality of life (∑ab = -0.09 [-0.27 – -0.02], β = -0.02).

**Conclusions:**

Our results suggest that adolescents’ emotion regulation has a prominent role in their quality of life in addition to attachment styles. To improve the quality of life among adolescents, we recommend using techniques that develop emotion regulation.

**Disclosure of Interest:**

None Declared

